# Mosaicism: Reason for Normal Phenotypes in Carriers of Small Supernumerary Marker Chromosomes With Known Adverse Outcome. A Systematic Review

**DOI:** 10.3389/fgene.2019.01131

**Published:** 2019-11-11

**Authors:** Thomas Liehr, Ahmed Al-Rikabi

**Affiliations:** Institute of Human Genetics, Jena University Hospital, Friedrich Schiller University, Jena, Germany

**Keywords:** small supernumerary marker chromosomes, genotype–phenotype correlation, Pallister–Killian syndrome, tetrasomy 9p, cat-eye syndrome, proximal tetrasomy 15q, isochromosome 18p

## Abstract

Small supernumerary marker chromosomes (sSMCs) are present in ∼3.3 million of presently living human beings. The majority of these sSMC carriers (i.e. ∼2.1 million) will never know about their condition, as they are perfectly healthy and just may learn by chance about it, e.g. if chromosomal analysis is done for some reason during their life time. The remainder ∼1.2 million of sSMC carriers are clinically affected either due to adverse effects of gained genetic material being present on the sSMC and/or by uniparental disomy of the sSMC’s sister chromosomes. Influence of mosaicism being present in 50% of sSMC carriers is controversy discussed in the literature. Even though genotype–phenotype correlation for sSMCs progressed during last years, still there are only eight sSMC-associated syndromes characterized yet, which may go together with mosaicism. Here we summarize presently available data for carriers of sSMCs normally leading to these well-defined syndromes, however, showing (almost) no clinical signs. This can be observed in ∼1 to 30% of the corresponding sSMC-carriers, thus, a high impact for counselling in corresponding prenatal *de novo* cases is not to be neglected.

## Introduction

Small supernumerary marker chromosomes (sSMCs) are at the same time structural chromosomal abnormalities as well as numerical ones (Liehr, 2012; Liehr, 2019). Thirty percent of sSMCs are inherited from a parent, while 70% are *de novo*. It is suggested that *de novo* sSMCs are products of trisomic rescue. The latter may be conveyed by different mechanisms, like U-type formation, ring chromosome-formation (Liehr, 2012; Liehr, 2019), or chromothripsis (Liehr, 2018; Kurtas et al., 2019). sSMCs are preferentially detected in three groups of patients: (i) infertile, (ii) patients with some kind of physical or mental impairment, and (iii) prenatally, in children with and without sonographic abnormalities. It can be estimated that in a world population of almost eight billion people, 3.3 million sSMC carriers should be present. Approximately 30% of sSMC carriers (1.2 million) are clinically impaired and may get the diagnoses to have an sSMC during life-time. Still most about 2.1 million of these extra chromosome carriers (70%) will never or only by chance learn about their condition (Liehr and Weise, 2007; Liehr, 2012).

An sSMC can derive from each of the 24 human chromosomes, can consist of continuous stretches of DNA from one or more chromosomes, can also be constituted from discontinuous parts of the same or different chromosomes, and contain hetero- and/or euchromatic DNA. Thus, especially for genetic counselling of prenatal *de novo* sSMC genotype–phenotype correlations are urgently needed. Research during last decades showed that there are two major players influencing clinical outcome: (a) gene content of the sSMC and (b) epigenetic influences mediated by imprinting (Liehr, 2012; Al-Rikabi et al., 2018). For (a) it is important to understand that only genes being dosage sensitive can have an impact on the sSMC carrier’s phenotype. Accordingly, sSMCs with euchromatin not necessarily are connected with adverse effects for its carrier, and it was already possible to characterize pericentric dosage-insensitive regions for each human chromosome (Al-Rikabi et al., 2018; Liehr, 2019). Presently, there are eight sSMC-related syndromes defined, which are due to adverse gene dosage effects, particularly partial tri- or tetrasomies; these are: isochromosome 5p- [Online Mendelian Inheritance in Man (OMIM) # n.a.], isochromosome 8p- (OMIM # n.a.), tetrasomy 9p- (OMIM # n.a.), proximal tetrasomy 15q- (OMIM # n.a.), Pallister–Killian (OMIM # 601803), isochromosome 18p- (OMIM #614290), isochromosome 20p- (OMIM # n.a.), and cat-eye-syndrome (OMIM #115470) (Liehr, 2019). Concerning (b) one must remember that *de novo* sSMCs normally derive from (incomplete) trisomic rescue. In most cases of any trisomy there are two copies of one maternal chromosome and one copy of a paternal one. In >95% of such cases where e.g. trisomic rescue is initiated one of the two maternal derived chromosomes is degraded. In the remainder cases the paternal copy is lost, which leads to a maternal uniparental disomy. The same may happen vice versa starting with two paternal chromosome copies in a trisomy. Especially if chromosomes 6, 7, 11, 14, 15, or 20 are concerned sSMC-presence may be a hint on an imprinting disease (Liehr et al., 2011; Liehr, 2012).

Another feature for sSMC carriers is that in 50% of the cases a mosaicism of cells with and without sSMC can be observed (Liehr et al., 2010). However, a human being comprises literally hundreds of different tissues while in diagnostics of a living person it is routine to study one, two maximally five different tissues only, for sSMC presence. Normally mosaicism as being observed in one tissue is suggested to be the approximate rate being present in all other tissues of this studied person (Liehr, 2012). Still, singular studies in aborted fetuses showed, that there is/maybe at least a substantial degree of variance in different tissues, and more important, that there is no obvious scheme behind the observable patterns. Particularly it is absolutely impossible to predict reliably the percentage of cells carrying an sSMC in the brain of a prenatally detected carrier by studying amnion-, chorion-, or even blood-cells (Fickelscher et al., 2007). Even though in the majority of the cases the presence of an sSMC known to be deleterious will lead to the expected adverse clinical outcome, during the last decade there were single case reports showing a normal or much less severe than to be expected outcome, especially in case of mosaicism (**Table 1**). Here we summarize these reports and estimate the frequency of clinically normal/only minor affected sSMC carriers in the eight sSMC-related syndromes listed in **Table 1**. Besides those eight sSMC-associated syndromes mentioned before there are three further syndromes being associated with so-called “complex sSMC” (Liehr et al., 2013); as these have a different mode of formation, never show mosaicism and also show no complete absence of phenotypes in sSMC carriers, Emanuel- (OMIM #609029), derivative chromosome 8 and 22- (OMIM #613700), and derivative chromosome 13/21 and 18-syndrome (OMIM # n.a.) were not included in this review.

**Table 1:** sSMC-associated syndromes, number of reported cases are given together with details on cases with no or minor phenotypical signs irrespective of deleterious sSMC and mosaicism with normal cells detected in studied tissues.

Case #	Tissue studied	sSMC %	Phenotype/Frequency
**Chromosome 5: isochromosome 5p-syndrome**		**27 cases reported**	**30%**
05-W-iso/1-13	CVS; AF; PBL	10/0/0	None
05-W-iso/1-14	CVS; AF; PBL	10/0/0	None
05-W-iso/1-15	CVS; AF; PBL	10/0/0	None
05-W-iso/1-16	CVS; AF; PBL	10/0/0	None
05-W-iso/1-17	CVS; AF; PBL	10/2/0	None
05-W-iso/1-18	CVS; AF; PBL	0/2/0	None
05-W-iso/1-19	PBL; skin; urine	16/0/0	INF
05-W-iso/1-23	AF/PBL; skin (normal); skin (hyperpigm.); urine; buccal mucosa	7/0/13/85/7/70	None
**Chromosome 8: isochromosome 8p-syndrome**		**23 cases reported**	**4%**
08-W-iso/2-1	PBL	70	None but dwarphism
**Chromosome 9: tetrasomy 9p-syndrome**		**107cases reported**	**8.4%**
09-W-iso/2-1	PBL; skin	16/0	None
09-W-iso/2-2	PBL; buccal mucosa	100/65	RAB
09-W-iso/2-3	PBL	47	INF
09-W-iso/2-4	PBL	n.a.	None
09-W-iso/2-5	PBL	72	INF
09-W-iso/3-1	PBL	100	Klinefelter like
09-W-iso/4-1	PBL; buccal mucosa	6/5	Klinefelter like
09-W-iso/4-2	PBL; buccal mucosa	100/85	None but dwarphism
09-W-iso/4-3	PBL; skin	30/0	None but dwarphism and Blashko lines
**Chromosome 12: Pallister–Killian syndrome**		**608 cases reported**	**0.8%**
12-Wpks-1	PBL; skin	0/37	Much less severe than normal PKS
12-Wpks-1a	PBL; skin	0/mosaic	Much less severe than normal PKS
12-Wpks-328	PBL; skin	0/mosaic	Much less severe than normal PKS
12-Wpks-329	Skin; buccal mucosa	mosaic/36%	Much less severe than normal PKS
12-Wpks-357b	PBL; buccal mucosa; hair root cells	50/0/0	None
**Chromosome 15: proximal tetrasomy 15q-syndrome**		**>1,000 cases reported**	**<0.7%**
15-O-q13/1-1	PBL	56	None
15-O-q13/1-2	AF (1); AF (2); PBL (birth); PBL (2y), PBL (4y)	23/6/26/46/36	None
15-O-q13/2-1	PBL	30	INF
15-O-q13/3-1	AF; PBL; skin; buccal mucosa	6/45/25/8	None
15-O-q13.1/1-1	AF/PBL derived cell line	79/61	None
Mother of 15-O-q13.1/1-1	PBL	10	None
15-O-q13.1/2-1	PBL	93	None
**Chromosome 18: isochromosome 18p-syndrome**		**320 cases reported**	**1.6%**
18-Wi-158	AF; PBL	35/0	None
18-Wi-158a	PBL	100	None
18-Wi-158b	AF	100	None
18-Wi-158c	AF (1); AF (2); PBL	21/14/0	None
18-Wi-272	PBL	11	Slight DD Much less severe than in i(18p) syndrome
**Chromosome 20: isochromosome 20p-syndrome**		**4 cases reported**	**0%**
n.a.	n.a.	n.a.	n.a.
**Chromosome 22: Cat-eye-syndrome**		**242 cases reported**	**3.2%**
22-Wces-5-168; father	PBL; buccal mucosa; spermatozoa	2.8/5.4/49.6	None
22-Wces-5-168; daughter 1	PBL; buccal mucosa	20/32	None
22-Wces-5-168; daughter “	PBL; buccal mucosa	29/63	Mild CES symptoms
22-Wces-5-168; son 1	PBL; buccal mucosa	27/47	Very minor CES symptoms
22-Wces-5-175	PBL	100	(None) No typical CES signs at all
22-Wces-5-192	PBL	100	(None) No typical CES signs at all
22-Wces-5-200	PBL	20	None
22-Wces-5-201	PBL	4.5	None

AF, amnion fluid; case #, identifier of the case acc. to Liehr (2019); CES, cat-eye-syndrome; CVS, chorion villi sampling; i(18p), isochromosome 18p-syndrome; INF, infertile; PBL, peripheral blood lymphocytes; PKS, Pallister–Killian syndrome; RAB, repeated abortions; sSMC %, percentage of cells with sSMC per tissue mentioned in column before. The frequency for (almost) normal phenotype for each of the 8 sSMC-associated syndromes is given in the column "Phenotype/ Frequency".

## Materials and Methods

### Literature Search

All reported sSMC cases are collected in the database: “Small supernumerary marker chromosomes” (accessible *via* http://ssmc-tl.com/sSMC.html, http://molbiol.sci.am/ssmc/ssmc-tl.com/sSMC.html or http://markerchromosomes.ag.vu/= Liehr, 2019). Cases reported with eight sSMC-related syndromes presenting with and without clinical symptoms were identified in this database and summarized in **Table 1**.

## Results

Overall, 48 cases out of 2,331 reported cases with sSMC-related syndromes (∼2%) showed (almost) normal outcomes, most likely due to mosaicism, reducing the normally adverse clinical signs and symptoms in parts to zero.

In seven out of eight sSMC-related syndromes cases without or only minor clinical symptoms were reported (**Table 1**). In isochromosome 20p-syndrome no clinically healthy sSMC carriers were identified, yet. For the remainder syndromes following percentages of clinically not or less affected than to be expected sSMC carriers were found (**Table 1**): isochromosome 5p-syndrome 30% (out of 27 cases), tetrasomy 9p-syndrome 8.4% (out of 107 cases), isochromosome 8p-syndrome 4% (out of 23 cases), cat-eye syndrome 3.2% (out of 242 cases), isochromosome 18p-syndrome 1.6% (out of 320 cases), Pallister–Killian syndrome 0.8% (out of 608 cases), and proximal tetrasomy 15q-syndrome <0.7% (out of >1,000 cases).

sSMC were found in different percentages of studied tissues of the tested persons listed in **Table 1**. Interestingly, there were several sSMC carriers without symptoms but 100% of cells with sSMC in peripheral blood lymphocytes, as observed in three cases with isochromosome 9p normally associated with tetrasomy 9p-syndrome, one case with isochromosome 18p-syndrome associated sSMC (plus 1 such case in amnion), and two cases with cat-eye syndrome-like sSMC.

## Discussion

sSMCs are a challenge especially for prenatal diagnostics and counselling. Here a yet underscored factor for predicting clinical outcome is reviewed, highlighted and discussed: the influence of mosaicism in cases with sSMC. As shown in a previous study for different tissues of an aborted sSMC carrier (Fickelscher et al., 2007) also the here summarized cases did not show any tendencies for defined mosaicism rates in different tissues (e.g. case 05-W-iso/1-23 with low rates of sSMC presence in amnion and blood but high rates in skin and urine, or case 22-Wces-5-168 with low sSMC rates in blood and buccal mucosa, but high rates in spermatozoa). Thus, general conclusions or clear predictions about grade of mosaicism in not studied tissues are not possible.

Frequencies of cases (almost) without clinical symptoms are different between the eight sSMC-associated syndromes (**Table 1**) and this has different reasons. No normal sSMC carriers were detected in isochromosome 20p-syndrome, which is most likely due to the small number of (i.e. only four) reported cases. For isochromosome 5p-syndrome 8 of 27 cases (30%) show normal outcomes. Here it must be considered also the small number of available reports, as well as the fact that six cases are prenatal ones from one single study with low rates of cells with isochromosome 5p in chorion or amnion (Venci and Bettio, 2009). Such cases may be more frequent for each numerical chromosomal abnormality, but normally are not reported in scientific papers (Yurov et al., 2018). Still there remain 2/21 cases (9.5%) with iso-chromosome 5p in adult without clinical symptoms, apart from infertility in one of the two cases. In isochromosome 8p-syndrome there is also necessary to consider the small number of reported cases, still 1 in 23 cases without symptoms gives a rate of 4%.

For remainder five other syndromes discussed here >100 case reports, and rates of <0.7 to 3.2% for proximal tetrasomy 15q-syndrome, cat-eye-syndrome, Pallister–Killian syndrome and isochromosome 18p-syndrome were determined, which are close to the overall 2% rate for normal outcomes in otherwise sSMC-related syndromes found here. Still the 8.4% rate for clinically (almost) healthy tetrasomy 9p-syndrome cases is remarkable, especially as this is the largest existent sSMC with overall 94.6 megabases of DNA being present as extra copy to the normal genetic content of a cell. For Pallister–Killian syndrome it must be admitted that for this condition mosaicism is rather the rule than exception, as the disease causing sSMC(12) is known to be lost in fast dividing tissues, regularly. However, patients still show the typical syndrome-associated clinical features. Accordingly, case 12-Wpks-357b with 50% of Pallister–Killian syndrome-typical sSMC in peripheral blood, but completely healthy, got the sSMC(12) restricted in him to peripheral blood most likely by fetal-fetal blood transfusion from his affected twin-sib, who had the sSMC in all body tissues. All other Pallister–Killian syndrome cases included in **Table 1** just show reduced but not completely absent symptoms.

The fact that 6 or 1/48 cases included here showed the sSMC in 100% of their peripheral blood cells or in 100% of amnion cells, is alarming. This means that among prenatal cases identified to be carriers of an sSMC known to be normally deleterious, there are ∼2% (for isochromosome 5p-, 8p-, and 9p-syndromes maybe much more) of such fetuses which have a normal clinical outcome.

Overall this review shows that somatic mosaicism being present in at least 50% of sSMC carriers is the third player besides genetic content and uniparental disomy influencing the clinical outcome. Even though overall only 2% of cases may be unexpectedly influenced positively by low mosaicism, e.g. in brain, this needs to be discussed in prenatal genetic counselling. Especially in case of isochromsomes 9, 8, and 5 this possibility could be even more important. Finally, in present times when main stream of human genetics promotes shifting all diagnostic efforts to high throughput approaches, it must be stressed here that (low-level) mosaicism like present in sSMC can only reliably be detected by single cell oriented approaches like banding and/or molecular cytogenetics.

## Data Availability Statement

The underlying datasets are available on http://ssmc-tl.com/sSMC.html, http://molbiol.sci.am/ssmc/ssmc-tl.com/sSMC.html and http://markerchromosomes.wg.am/.

## Author Contributions

TL drafted the paper, and did the literature search and development of discussion part together with AA-R.

## Conflict of Interest

The authors declare that the research was conducted in the absence of any commercial or financial relationships that could be construed as a potential conflict of interest.
